# Supplementary figures and images for: Rapid restructurization of conformationally-distinct alpha-synuclein amyloid fibrils at an elevated temperature
Source: PeerJ. 2022 Sep 30;10:e14137. doi: 10.7717/peerj.14137 (PMC9528901; doi:10.7717/peerj.14137)

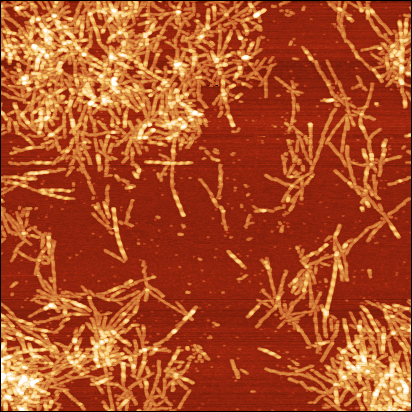

Supplement: Supplemental Information 6 [file peerj-10-14137-s006.zip › Type 1 AFM 0h.tiff]

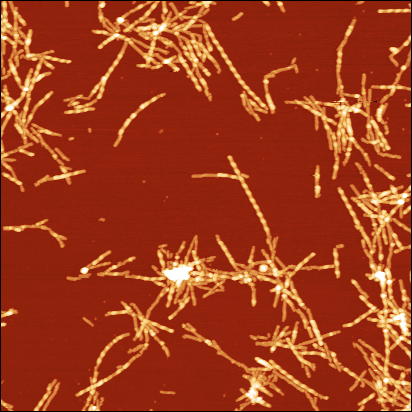

Supplement: Supplemental Information 6 [file peerj-10-14137-s006.zip › Type 1 AFM 48h.tiff]

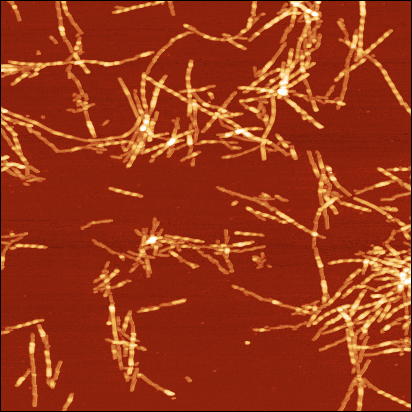

Supplement: Supplemental Information 6 [file peerj-10-14137-s006.zip › Type 2 AFM 0h.tiff]

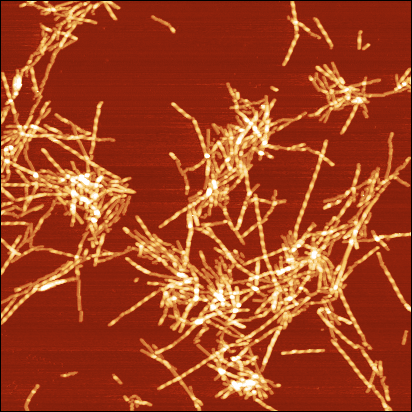

Supplement: Supplemental Information 6 [file peerj-10-14137-s006.zip › Type 2 AFM 48h.tiff]

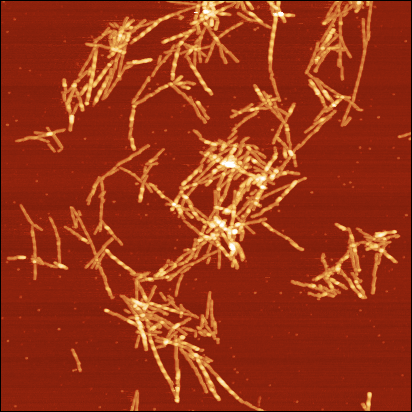

Supplement: Supplemental Information 6 [file peerj-10-14137-s006.zip › Type 3 AFM 0h.tiff]

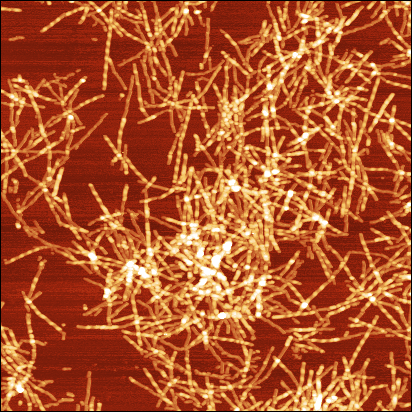

Supplement: Supplemental Information 6 [file peerj-10-14137-s006.zip › Type 3 AFM 48h.tiff]

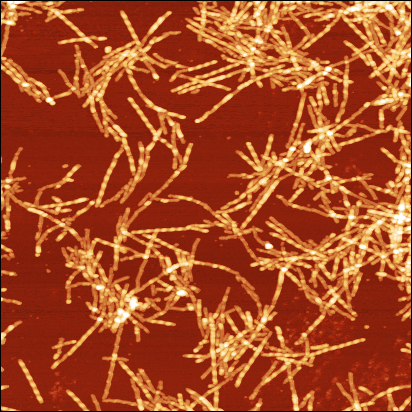

Supplement: Supplemental Information 6 [file peerj-10-14137-s006.zip › Type 4 AFM 48h.tiff]

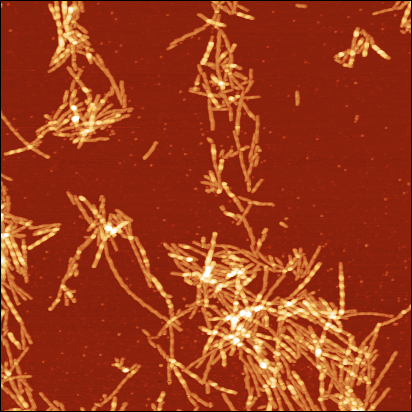

Supplement: Supplemental Information 6 [file peerj-10-14137-s006.zip › Type 4 AFM 0h.tiff]
